# A high triglyceride glucose index is more closely associated with hypertension than lipid or glycemic parameters in elderly individuals: a cross-sectional survey from the Reaction Study

**DOI:** 10.1186/s12933-020-01077-6

**Published:** 2020-07-14

**Authors:** Binruo Zhu, Jie Wang, Kang Chen, Wenhua Yan, Anping Wang, Weiqing Wang, Zhengnan Gao, Xulei Tang, Li Yan, Qin Wan, Zuojie Luo, Guijun Qin, Lulu Chen, Yiming Mu

**Affiliations:** 1 Medicine School of Nankai University, Tianjin, China; 2 Department of Endocrinology, Chinese PLA General Hospital, 28 Fu Xing Road, Beijing, 100853 People’s Republic of China; 3 Shanghai National Research Centre for Endocrine and Metabolic Diseases, State Key Laboratory of Medical Genomics, Shanghai Institute for Endocrine and Metabolic Diseases, Ruijin Hospital, Shanghai Jiaotong University School of Medicine, Shanghai, China; 4 Dalian Central Hospital, Dalian, Liaoning China; 5 First Hospital of Lanzhou University, Lanzhou, Gansu China; 6 Zhongshan University Sun Yat-sen Memorial Hospital, Guangzhou, Guangdong China; 7 Southwest Medical University Affiliated Hospital, Luzhou, Sichuan China; 8 First Affiliated Hospital of Guangxi Medical University, Nanning, Guangxi China; 9 First Affiliated Hospital of Zhengzhou University, Zhengzhou, Henan China; 10 Wuhan Union Hospital, Huazhong University of Science and Technology, Wuhan, Hubei China

**Keywords:** TyG index, HTN, Lipid parameters, Glycemic parameters

## Abstract

**Background:**

Both lipid and glucose abnormalities are associated with hypertension (HTN). However, it is unclear whether the triglyceride-glucose (TyG) index is associated with HTN. Therefore the aim of this study is to investigate the association of the TyG index and HTN and to compare the discriminative power of the TyG index, lipid, glycemic parameters for the risk of HTN in elderly individuals.

**Methods:**

The present study was nested in a longitudinal (REACTION) study from May 2011 to December 2011, which was designed to demonstrate the association of abnormal glucose metabolism with the risk of cancer in the Chinese population. In total, 47,808 participants were recruited in this cross-sectional study. The TyG index was divided into five groups: the < 20% group, the 20–39% group, the 40–59% group, the 60–79% group and the ≥ 80% group, according to quintile division of the subjects. Three multivariate logistic regression models were used to evaluate the association between the TyG vs. lipid parameters, glycemic parameters and HTN.

**Results:**

Multivariate logistic regression analysis shows that compared with lipid and glycemic parameters, the TyG index remains significantly associated with HTN in either total subjects or subjects separated into men and women (odds ratio (OR) 1.33, 95% confidence interval (CI) 1.18–1.51, p < 0.0001 in total subjects; OR 1.39, 95% CI 1.11–1.74, p = 0.0042 in men; OR 1.28, 95% CI 1.11–1.49, p = 0.0010 in women). In a stratified analysis, an elevated TyG index is significantly associated with HTN in the subgroup of the oldest age (≥ 65) (OR 1.67, 95% CI 1.30–2.14, p < 0.0001), as well as with obesity (Body mass index (BMI) ≥ 28 kg/m^2^) (OR 1.85, 95% CI 1.29–2.66, p = 0.0009) or lower estimated glomerular filtration rate (eGFR) (< 90 mL/(min·1.73 m^2^)) (OR 1.72, 95% CI 1.33–2.21, p < 0.0001).

**Conclusion:**

The TyG index is significantly associated with HTN and shows the superior discriminative ability for HTN compared with lipid and glycemic parameters in the Chinese elderly population.

## Background

Hypertension (HTN) is one of the most prevalent cardiovascular risk factors, with over 34% of males and 28% of women aged ≥ 25 years being affected globally by raised blood pressure [1]. With 20% of the world’s population, China represents a large portion of this burden, where HTN and blood pressure-related cardiovascular diseases (CVDs) are major public health challenges [2, 3]. HTN prevalence has risen in recent decades, resulting in an increase of blood pressure-related morbidity and mortality.

It is well known that both lipid and glucose abnormalities are associated with HTN. It is reported that dyslipidemia has been observed in 50% to 80% of hypertensive patients [4]. Dyslipidemia, comprising elevated triglyceride (TG), high cholesterol (TC), increased low-density lipoprotein cholesterol (LDL-C), and decreased high-density lipoprotein cholesterol (HDL-C), is independently associated with HTN or other CVDs risk factors [5–8]. There are renewed interests engendered by epidemiological and genetic evidence proving that increased TG, remnant TC, or TG-rich lipoproteins are additional causes of CVDs and all-cause mortality [9]. Similarly, HTN and type 2 diabetes (T2DM) are common causes of morbidity; both constitute risk factors for CVDs and might be engaged in similar genetic and environmental risk factors [10]. It is reported that elevated plasma glucose is a steady and independent predictor of HTN [11]. Some modern antidiabetic drugs are also capable of lowering both office and ambulatory blood pressure. This can contribute to the favorable effect on some clinical endpoints, most importantly the reduction of congestive heart failure and cardiovascular mortality [12, 13].

The main pathogenetic pathways linking T2DM, dyslipidemia and HTN are thought to be through insulin resistance (IR) and increased activity of the sympathetic nervous system and the renin–angiotensin–aldosterone system as well as increased renal sodium reabsorption [14]. The association between IR and the risk of incident HTN was shown in a recent meta-analysis of 11 studies [15], suggesting that IR could be employed as an adjunctive tool to identify individuals at potential risk for HTN. Glucose clamp technique is the gold standard for IR measurement initially proposed by De Fronzo [16]. However, such direct diagnostic tests have considerably high costs and low availability for epidemiologic use [17].

In recent years, the triglyceride-glucose (TyG) index is arising as an ideal substitution for IR [18, 19]. It is calculated as ln [fasting plasma glucose (FBG)(mg/dL) * TG (mg/dL)/2] [20]. This measurement merely requires simple lab tests like TG and plasma glucose, which can be obtained in highly cost-effective and time-efficient ways. Additionally, the TyG index has been revealed to determine IR in a more appropriate way than other substitutional indexes like HOMA-IR, which was compared with the gold standard method for IR [21]. Previous studies showed that the TyG index is closely associated with HTN [22], artery stiffness and coronary artery calcification [23–25]. Furthermore, the TyG index can predict coronary artery disease severity and cardiovascular outcomes [26]. The association between the TyG index and T2DM was also demonstrated in Spain, China and Korea [19, 27–29].

However, studies are limited that involve the association between the TyG index and HTN and comparison of the discriminative abilities of the TyG index, lipid, glycemic parameters for the risk of HTN. Therefore our study intends to explore the association of the TyG index with HTN and compare the discriminative power of the TyG index, lipid, glycemic parameters for HTN in elderly individuals in China.

## Methods

### Study subjects

**Fig. 1:**
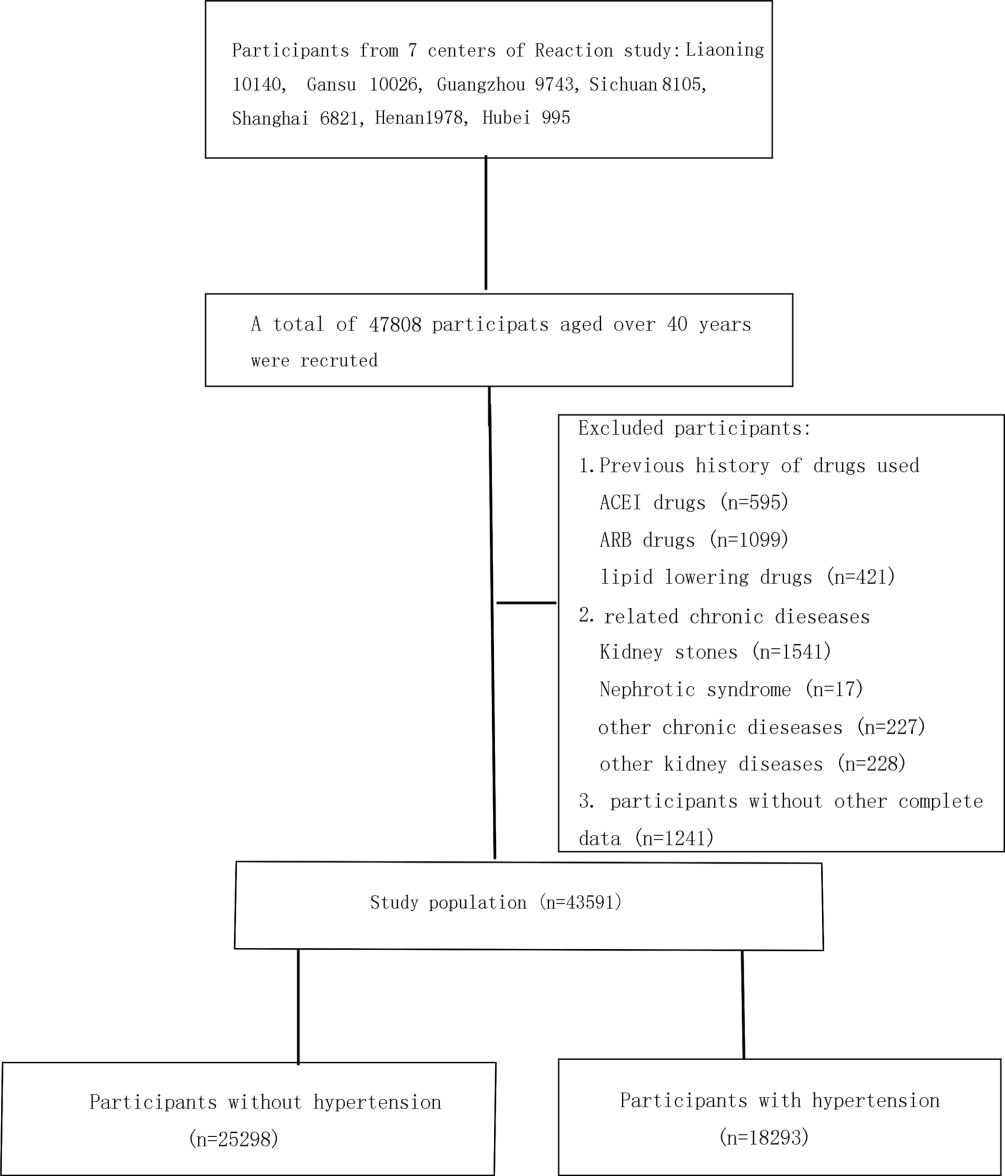
Flow chart of the selection of study participants

The present study assessed 47,808 participants aged over 40 years from a longitudinal REACTION study (Risk Evaluation of cAncers in Chinese diabeTic Individuals), including seven regional centers (Gansu, Guangdong, Henan, Hubei, Liaoning, Shanghai, and Sichuan), from May 2011 to December 2011. Previous history of related chronic diseases, using ACEI/ARB medicines, lipid-lowering drugs, missed data and/or included outliers, were exclusion criteria in the study. Finally, 43,591 participants were recruited (Fig. 1).

Before the investigation, the clinicians were well-trained for the questionnaire and data collection. The present study was approved by the Committee on Human Research at Rui-Jin Hospital affiliated with the School of Medicine, Shanghai Jiao Tong University and all participants recruited had signed informed consents before data collection.

### Clinical data and biochemical indicators

The subjects received the following examinations: a standardized questionnaire, anthropometric measurements, blood collection, and a standard 75-goral glucose tolerance test (OGTT) or standard meal test. The same trained clinicians carried out standard questionnaires, which included demography, lifestyle, history of diabetes, stroke, coronary heart disease (CHD), HTN and dyslipidemia as well as the medication history, including the use of drugs. All data were maintained corresponding to established standard methods by the same well-trained clinicians. Physical examination items included height, weight, waist circumference, hip circumference, blood pressure, and heart rate. Height was measured in bare feet accurate to 0.01 m. Weight was measured in light clothes accurate to 0.1 kg. Waist circumference and hip circumference were measured to an accuracy of 0.01 m by the same staff. Waist circumference/hip circumference (WHR) was accurate to 0.01. Body mass index (BMI) was calculated as weight/height^2^. After at least a 5-minute rest, blood pressure was measured seated three times with an interim of 1 min, using an OMRON electronic blood pressure monitor. The average blood pressure was calculated and used for analysis. The estimated glomerular filtration rate (eGFR) was determined by the modified MDRD equation [30]: eGFR = 186 × (serum creatinine × 0.011) −1.154 × (age)^−0.203^ × (0.742 if female) × 1.233. TyG (mg/dl)^2^ = ln [FBG (mg/dl) * TG (mg/dl)/2].

### 75 g OGTT or standard meal test

After an overnight fast for at least 12 h, the first fasting blood samples were obtained for FBG measurement. Standard 75 g glucose solution was given to the individuals without a T2DM history, while standard meals containing 100 g carbohydrates were given to the individuals with a T2DM history. Blood samples for glucose measurement were obtained at 120 min after either 75 g OGTT or standard meal test. FBG and 2 h post-load blood glucose (PBG) were measured by the Hexokinase method on an autoanalyzer.

TG, TC, HDL-C, LDL-C, alanine transferase (ALT), aspartate transferase (AST), serum creatinine (Cr) and gamma-glutamyl transferase (GGT) were measured by chemiluminescence on an autoanalyzer. Glycosylated hemoglobin (HbA1c) was measured by high pressure liquid chromatography.

### Definition of variables

TyG index was divided into five groups: the < 20% group, the 20–39% group, the 40–59% group, the 60–79% group and the ≥ 80% group, according to quintile division of the subjects. As the Chinese guideline for the management of dyslipidemia in adults (revised in 2016) [31] suggests, lipid parameters were categorized as follows: 1. TG: normal: < 1.7 mmol/L, borderline high: 1.7–2.3 mmol/L, high: ≥ 2.3 mmol/L; 2. TC: normal: < 5.2 mmol/L, borderline high: 5.2–6.2 mmol/L, high: ≥ 6.2 mmol/L; 3. HDL-C: low risk: ≥ 1.0 mmol/L, high risk: < 1.0 mmol/L; 4. LDL-C: ideal < 2.6 mmol/L, borderline high: 3.4–4.1 mmol/, high: ≥ 4.1 mmol/L. According to the WHO guidelines, T2DM was defined as FBG ≥ 7.0 mmol/L, or PBG ≥ 11.1 mmol/L, or a self-reported history of T2DM. HbA1c was divided into two groups according to the American Diabetes Association [32] and the World Health Organization [33]: normal: < 6.5% and high ≥ 6.5%. HTN was defined as systolic blood pressure (SBP) ≥ 140 mmHg or diastolic blood pressure (DBP) ≥ 90 mmHg, or diagnosed as hypertension by clinicians and meanwhile undergoing antihypertensive-medication therapy. The subjects who were once diagnosed as hypertension but present status could not meet diagnostic criteria were treated as normotensive in present study. Participants were divided into three groups according to their smoking frequency: no: never or have already quit smoking; occasional: smoking less than once a week or less than 7 cigarettes weekly; frequently: smoking one or more cigarettes daily for at least a half year. Similarly, participants were divided into three groups according to their alcohol intake frequency: no: never or have already quit drinking; occasional: drinking less than once a week; frequently: drinking more than once a week for at least a half year. Stroke, including all subtypes, was determined according to a subject’s self-report, including a history of language or physical dysfunction lasting over 24 h and ischemic or hemorrhagic stroke by imageological diagnosis. CHD events were defined as any self-report history of hospital-admitted myocardial infarction or angina, or coronary revascularization. CVDs were also screened according to a subject’s self-report, including history of CHD, stroke, or myocardial infarction.

### Statistical analysis

Empower(R) (www.empowerstats.com, X&Y Solutions Inc., Boston, MA) and R (http://www.Rproject.org) were employed to perform the statistical analyses. The odds ratio (OR) and the 95% confidence intervals (CI) were calculated. P values < 0.05 (2-sided) were considered to indicate statistical significance.

Variables were presented as mean ± standard deviation (SD), if normal distribution. And if they were not, they were presented as median (Q1-Q3), or n (%). The differences between continuous variables were compared using the Kruskal–Wallis test. The percentage difference between groups was compared using the χ^2^ test. Three multivariate logistic regression models were built to identify the associations between TyG vs. lipid parameters, PBG, HbA1c and HTN. Model 0 was not adjusted for any confounding factors, while Model 1 was adjusted for age and sex. Model 2 was further adjusted for age; center; sex; history of CVDs; history of T2DM; hypoglycemic drugs; SBP; DBP; BMI; ALT; AST; WHR; eGFR; smoking habits and drinking habits. Stratified analyses were conducted by the different level of age (G1: < 55, G2: 55–65, G3: ≥ 65), BMI (underweight: < 18.5 kg/m^2^; normal weight: 18.5–24 kg/m^2^; overweight: 24–28 kg/m^2^, obesity: BMI of ≥ 28 kg/m^2^, according to Cooperative Meta-analysis Group of China Obesity Task Force report [34]) and eGFR (G1: < 90 mL/(min·1.73 m^2^), G2: ≥ 90 mL/(min·1.73 m^2^), according to KDIGO [35]). Subjects were stratified into subgroups to separately explore the relevant underlying factors which might affect the relationship between the TyG index and HTN. Meanwhile, potential interactions of the TyG index and strata variables were assessed in the logistical regression analysis.

## Results

### Characteristics of study population by HTN

The study included 43,591 participants (Table 1), of whom 18,293 (40.5%) had HTN. Compared to non-hypertensive participants, the hypertensive ones were older, possessed a larger BMI, were less frequent smokers, less frequent drinkers, had a higher mean SBP and DBP, a higher heart rate, a less favorable metabolic profile (FBG, PBG, HbA1c, AST, ALT, GGT, TC, TG), lower levels of eGFR, and a higher frequency of CVDs and T2DM.

**Table 1:** Characteristics of study population by HTN

	No HTN	HTN	*P* value
N	25,298	18,293	
Age, years	55.41 (50.05–60.73)	60.89 (55.38–68.15)	< 0.001
BMI, kg/m^2^	23.46 (21.47–25.67)	25.33 (23.23–27.64)	< 0.001
SBP, mmHg	121.00 (112.00–130.00)	149.00 (140.00–162.00)	< 0.001
DBP, mmHg	73.00 (68.00–79.00)	85.00 (77.00–92.00)	< 0.001
HR	78.00 (71.00–86.00)	79.00 (72.00–88.00)	< 0.001
FBG, mmol/L	5.40 (5.02–5.91)	5.77 (5.29–6.58)	< 0.001
PBG, mmol/L	7.00 (5.81– 8.84)	8.29 (6.60–11.24)	< 0.001
HbA1c, %	5.80 (5.60–6.20)	6.00 (5.70–6.50)	< 0.001
ALT, U/L	14.00 (10.00–20.00)	16.00 (12.00–22.00)	< 0.001
AST, U/L	20.00 (16.00–24.00)	21.00 (17.00–25.00)	< 0.001
GGT, U/L	19.00 (14.00–28.00)	23.00 (16.00–35.00)	< 0.001
TG, mmol/L	1.24 (0.90–1.79)	1.52 (1.08–2.17)	< 0.001
TC, mmol/L	4.93 (4.21–5.68)	5.14 (4.38–5.88)	< 0.001
LDL-C, mmol/L	2.84 (2.28–3.45)	2.99 (2.40–3.61)	< 0.001
HDL-C, mmol/L	1.31 (1.09–1.55)	1.25 (1.06–1.47)	< 0.001
eGFR, mL/(min·1.73 m^2^)	96.85 (93.10–100.56)	92.98 (88.31–96.85)	< 0.001
Sex, (%)			< 0.001
Male	6918 (27.35%)	6201 (33.90%)	
Female	18,380 (72.65%)	12,092 (66.10%)	
Drinking, (%)			< 0.001
Never	18,643 (73.69%)	14,023 (76.66%)	
Occasional	5278 (20.86%)	2883 (15.76%)	
Frequently	1377 (5.44%)	1387 (7.58%)	
Smoking, (%)			< 0.001
Never	21,443 (84.76%)	15,779 (86.26%)	
Occasional	862 (3.41%)	495 (2.71%)	
Frequently	2993 (11.83%)	2019 (11.04%)	
CVDs, (%)			< 0.001
Yes	681 (2.69%)	1690 (9.24%)	
No	24,617 (97.31%)	16,603 (90.76%)	
T2DM, (%)			< 0.001
No	22,723 (89.82%)	14,226 (77.77%)	
Yes	2575 (10.18%)	4067 (22.23%)	

Data were mean ± SD or median (IQR) for skewed variables or numbers (proportions) for categorical variables

*HTN* hypertension, *BMI* body mass index, *SBP* systolic blood pressure, *DBP* diastolic blood pressure, *HR* heart rate, *FBG* fasting plasma glucose, *PBG* 2 h post-load blood glucose, *HbA1c* glycosylated hemoglobin, *ALT* alanine transferase, *AST* aspartate transferase, *GGT* gamma-glutamyl transferase, *TG* triglyceride, *TC* high cholesterol, *LDL*-*C* low-density lipoprotein cholesterol, *HDL*-*C* high-density lipoprotein cholesterol, *eGFR* estimated glomerular filtration rate, *CVD* cardiovascular disease, *T2DM* type 2 diabetes

### Association of the TyG index, glycemic, lipid parameters with HTN

**Fig. 2:**
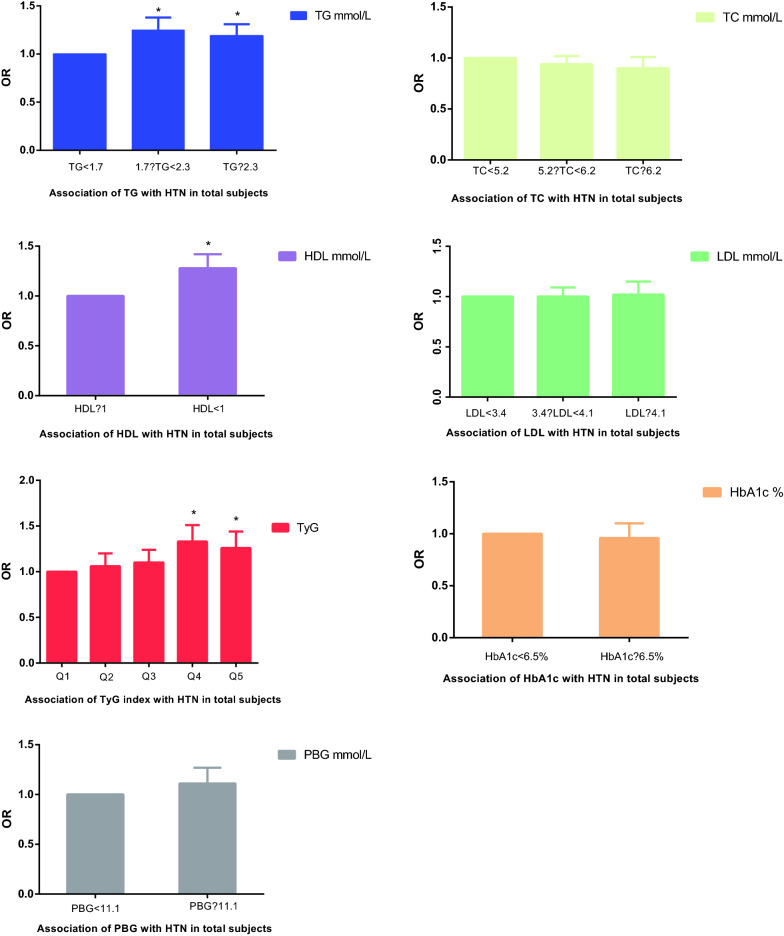
Association of the TyG index, glycemic, lipid parameters with HTN in total subjects in Model II (adjusted for age; center; history of CVDs; history of T2DM; hypoglycemic drugs; SBP; DBP; BMI; ALT; AST; WHR; eGFR; smoking habits; drinking habits. P < 0.05*)

Multiple logistic regression models that consider separately each index and their individual components as predictors of HTN were constructed. Table 2 shows OR and 95% CI of HTN with the groups of TG, TC, HDL-C, LDL-C, TyG quintiles, HbA1c and PBG in the total population within three different models. As seen in Table 2, every index is significantly associated with HTN in the non-adjusted model. However, after further adjustments in Model II, only the fourth and fifth quintiles of TyG (fourth quintile: OR 1.33, 95% CI 1.18–1.51, p < 0.0001; fifth quintile: OR 1.26, 95% CI 1.11–1.44, p = 0.0005), HDL-C ≥ 1 mmol/L (OR 1.28, 95% CI 1.15–1.42, P = 0.0005), TG ≥ 1.7 mmol/L (1.7 ≤ TG < 2.3 mmol/L: OR 1.25, 95% CI 1.13–1.38, P < 0.0001; TG ≥ 2.3 mmol/L: OR 1.19, 95% CI 1.07–1.31, P = 0.0007) and PBG (OR 1.02, 95% CI 1.00–1.03, p = 0.0117) remained significantly associated with HTN, whereas TC, HDL-C, LDL-C, HbA1c were not. The above results are displayed as a color-coded figure shown in Fig. 2. The association between hypertension and continuous values of the TyG index, glycemic, lipid parameters is shown in Additional File 1 Table S1, which was consistent to the finding delivered by categorical values. No effect of interaction between FBG and TG was observed after adjustment as shown in Additional file Table S2.

**Table 2:** Association of the TyG index, glycemic, lipid parameters with HTN in total subjects

Variable	Non-adjusted		Adjust I		Adjust II	
	OR (95% CI)	P-value	OR (95% CI)	P-value	OR (95% CI)	P-value
TG, mmol/L						
< 1.7	1.0		1.0		1.0	
≥ 1.7, < 2.3	1.72 (1.63, 1.81)	< 0.0001	1.63 (1.55, 1.72)	< 0.0001	1.25 (1.13, 1.38)	< 0.0001
≥ 2.3	1.99 (1.89, 2.10)	< 0.0001	2.01 (1.91, 2.12)	< 0.0001	1.19 (1.07, 1.31)	0.0007
TC, mmol/L						
< 5.2	1.0		1.0		1.0	
≥ 5.2, < 6.2	1.28 (1.23, 1.34)	< 0.0001	1.25 (1.19, 1.31)	< 0.0001	0.94 (0.86, 1.02)	0.1432
≥ 6.2	1.46 (1.39, 1.55)	< 0.0001	1.39 (1.31, 1.48)	< 0.0001	0.90 (0.81, 1.01)	0.0620
HDL-C, mmol/L						
≥ 1	1.0		1.0		1.0	
< 1	1.18 (1.13, 1.25)	< 0.0001	1.15 (1.09, 1.22)	< 0.0001	1.28 (1.15, 1.42)	< 0.0001
LDL-C, mmol/L						
< 3.4	1.0		1.0		1.0	
≥ 3.4, < 4.1	1.31 (1.25, 1.37)	< 0.0001	1.24 (1.18, 1.31)	< 0.0001	1.00 (0.91, 1.09)	0.9455
≥ 4.1	1.48 (1.39, 1.57)	< 0.0001	1.39 (1.30, 1.48)	< 0.0001	1.02 (0.90, 1.15)	0.7711
TYG in transform Q5						
Q1	1.0		1.0		1.0	
Q2	1.54 (1.44, 1.64)	< 0.0001	1.41 (1.31, 1.51)	< 0.0001	1.06 (0.94, 1.20)	0.3362
Q3	2.02 (1.89, 2.15)	< 0.0001	1.76 (1.64, 1.88)	< 0.0001	1.10 (0.98, 1.24)	0.1173
Q4	2.80 (2.62, 2.98)	< 0.0001	2.36 (2.21, 2.53)	< 0.0001	1.33 (1.18, 1.51)	< 0.0001
Q5	3.79 (3.55, 4.04)	< 0.0001	3.32 (3.10, 3.55)	< 0.0001	1.26 (1.11, 1.44)	0.0005
HbA1c, %						
< 6.5	1.0		1.0		1.0	
≥ 6.5	2.19 (2.08, 2.30)	< 0.0001	1.76 (1.67, 1.85)	< 0.0001	0.96 (0.84, 1.10)	0.5782
PBG, mmol/L						
< 11.1	1.0		1.0		1.0	
≥ 11.1	2.40 (2.28, 2.52)	< 0.0001	1.94 (1.84, 2.04)	< 0.0001	1.11 (0.96, 1.27)	0.1535

Model 0: Adjusted for no confounding factors

Model 1: Adjusted for age and gender

Model 2: age; center; sex; history of CVDs; history of T2DM; hypoglycemic drugs; SBP; DBP; BMI; ALT; AST; WHR; eGFR; smoking habits; drinking habits

**Fig. 3:**
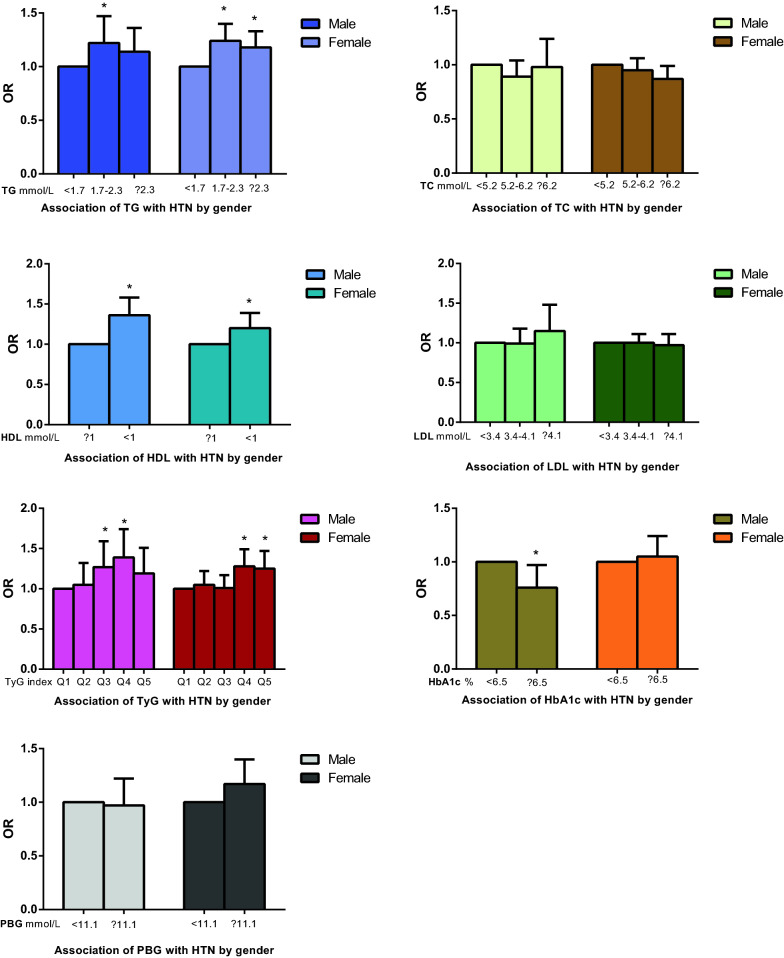
Association of the TyG index, glycemic, lipid parameters with HTN by gender in Model II (adjusted for age; center; history of CVDs; history of T2DM; hypoglycemic drugs; SBP; DBP; BMI; ALT; AST; WHR; eGFR; smoking habits; drinking habits. P < 0.05*)

Table 3 shows similar results in subjects separated into men and women: (1) male: third and fourth quintiles of TyG (third quintile: OR 1.27, 95% CI 1.02–1.59, p = 0.0309; fourth quintile: OR 1.39, 95% CI 1.11–1.74, p = 0.0042), HDL-C (OR 1.36, 95% CI 1.16–1.58, p < 0.0001) and TG ≥ 1.7 mmol/L (1.7 ≤ TG<2.3 mmol/L: OR 1.22, 95% CI 1.02–1.47, p = 0.0336) are all associated with HTN. To be noted, a slight negative association between HbA1c and HTN was revealed in the male population (OR 0.76, 95% CI 0.59–0.97, p = 0.0292). (2) female: fourth and fifth quintiles of TyG (fourth quintile: OR 1.28, 95% CI 1.11–1.49, p = 0.0010; fifth quintile: OR 1.25, 95% CI 1.07–1.47, p = 0.0057), HDL-C (OR 1.20, 95% CI 1.04–1.39, p < 0.0001), TG ≥ 1.7 mmol/L (1.7 ≤ TG < 2.3: OR 1.24, 95% CI 1.10–1.40, p = 0.0003; TG ≥ 2.3 mmol/L: OR 1.18, 95% CI 1.04–1.33, p = 0.0098) and PBG (OR 1.03, 95% CI 1.01–1.05, p = 0.0023) are associated with HTN. The above results are displayed as a color-coded figure shown in Fig. 3.

**Table 3:** Association of the TyG index, glycemic, lipid parameters with HTN by sex in Model 2

Variable	Male		Female	
	OR (95% CI)	P-value	OR (95% CI)	P-value
TG, mmol/L				
< 1.7	1.0		1.0	
≥ 1.7, < 2.3	1.22 (1.02, 1.47)	0.0336	1.24 (1.10, 1.40)	0.0003
≥ 2.3	1.14 (0.96, 1.36)	0.1377	1.18 (1.04, 1.33)	0.0098
TC, mmol/L				
< 5.2	1.0		1.0	
≥ 5.2, < 6.2	0.89 (0.76, 1.04)	0.1454	0.95 (0.86, 1.06)	0.3689
≥ 6.2	0.98 (0.78, 1.24)	0.8749	0.87 (0.77, 0.99)	0.0289
HDL-C, mmol/L				
≥ 1	1.0		1.0	
< 1	1.36 (1.16, 1.58)	< 0.0001	1.20 (1.04, 1.39)	0.0128
LDL-C, mmol/L				
< 3.4	1.0		1.0	
≥ 3.4, < 4.1	0.99 (0.83, 1.18)	0.9088	1.00 (0.89, 1.11)	0.9433
≥ 4.1	1.15 (0.89, 1.48)	0.2941	0.97 (0.84, 1.11)	0.6651
TYG ln transform Q5				
Q1	1.0		1.0	
Q2	1.05 (0.85, 1.32)	0.6358	1.05 (0.91, 1.22)	0.4763
Q3	1.27 (1.02, 1.59)	0.0309	1.01 (0.88, 1.17)	0.8498
Q4	1.39 (1.11, 1.74)	0.0042	1.28 (1.11, 1.49)	0.0010
Q5	1.19 (0.95, 1.51)	0.1376	1.25 (1.07, 1.47)	0.0057
HbA1c, %				
< 6.5	1.0		1.0	
≥ 6.5	0.76 (0.59, 0.97)	0.0292	1.05 (0.89, 1.24)	0.5480
PBG, mmol/L				
< 11.1	1.0		1.0	
≥ 11.1	0.97 (0.77, 1.22)	0.7897	1.17 (0.98, 1.40)	0.0798

Model 2: Adjusted for age; center; history of CVDs; history of T2DM; hypoglycemic drugs; SBP; DBP; BMI; ALT; AST; WHR; eGFR; smoking habits; drinking habits

### Associations between the TyG index and HTN in individuals with LDL-C < 2.6 mmol/L or HDL-C > 1.0 mmol/L

As the Chinese guideline for the management of dyslipidemia in adults (revised in 2016) [31] suggests, the population was categorized into two groups, LDL-C < 2.6 mmol/L (ideal value) and HDL-C ≥ 1.0 mmol/L (low risk value). As Table 4 shows, high TyG levels (the fourth and fifth quantile) were still significantly associated with HTN even when LDL-C or HDL-C was well-controlled (When LDL-C was well-controlled: the forth quintile of TyG: OR 1.26, 95% CI 1.10–1.44, p = 0.0026; the fifth quintile of TyG: OR 1.24, 95% CI 1.07–1.44, p = 0.0080. When HDL-C was well-controlled: the forth quintile of TyG: OR 1.28, 95% CI 1.09–1.50, p = 0.0007; the fifth quintile of TyG: OR 1.27, 95% CI 1.06–1.51, p = 0.0026). Moreover, medium and high TG levels (≥ 1.7 mmol/L) as well as high HDL-C levels (≥ 1 mmol/L) also remained associated with HTN when LDL-C or HDL-C was well-controlled. The associations were not statistically significant in PBG, HbA1c or other lipid parameters.

**Table 4:** Associations between the TyG index and HTN in people with LDL-C < 2.6 mmol/L or HDL-C > 1.0 mmol/L

Variable	HDL-C > 1.0 mmol/L		LDL-C < 2.6 mmol/L	
	OR (95% CI)	P-value	OR (95% CI)	P-value
TG, mmol/L				
< 1.7	1.0		1.0	
≥ 1.7, < 2.3	1.24 (1.11, 1.39)	0.0001	1.19 (1.06, 1.34)	0.0040
≥ 2.3	1.19 (1.05, 1.33)	0.0048	1.12 (0.99, 1.28)	0.0772
TC, mmol/L				
< 5.2	1.0		1.0	
≥ 5.2, < 6.2	0.97 (0.88, 1.06)	0.4546	0.99 (0.89, 1.10)	0.8933
≥ 6.2	0.94 (0.83, 1.05)	0.2736	0.96 (0.85, 1.09)	0.5788
HDL-C, mmol/L				
≥ 1	–		1.0	
< 1	–		1.26 (1.05, 1.50)	0.0125
LDL-C, mmol/L				
< 3.4	1.0		–	
≥ 3.4, < 4.1	1.00 (0.90, 1.11)	0.9993	–	
≥ 4.1	1.04 (0.92, 1.18)	0.5327	–	
TYG ln transform Q5				
Q1	1.0		1.0	
Q2	1.03 (0.90, 1.17)	0.6633	1.09 (0.93, 1.29)	0.2728
Q3	1.05 (0.92, 1.20)	0.4438	1.12 (0.95, 1.31)	0.1734
Q4	1.26 (1.10, 1.44)	0.0007	1.28 (1.09, 1.50)	0.0026
Q5	1.24 (1.07, 1.44)	0.0042	1.27 (1.06, 1.51)	0.0080
HbA1c, %				
< 6.5	1.0		1.0	
≥ 6.5	1.01 (0.87, 1.18)	0.8694	0.96 (0.78, 1.20)	0.7372
PBG, mmol/L				
< 11.1	1.0		1.0	
≥ 11.1	1.13 (0.97, 1.31)	0.1196	1.22 (0.98, 1.50)	0.0690

Adjusted for age; center; sex; history of CVDs; history of T2DM; hypoglycemic drugs; SBP; DBP; BMI; ALT; AST; WHR; eGFR; smoking habits; drinking habits

### Associations between the TyG index and HTN for stratified subgroups of age, BMI and eGFR

Stratified analyses were conducted in the different subgroups to further validate the abovementioned results, shown in Table 5. The present study suggested that compared with participants with lower TyG levels, subjects with higher TyG levels (the fourth and fifth quintile) were more closely associated with HTN in the older age (≥ 55 years), higher level of BMI (≥ 24 kg/m^2^) and both eGFR subgroups. To be noted, these associations were most significant in the subjects that were both in the subgroup of the forth quintile of TyG and the subgroup of oldest age (≥ 65 years) (OR 1.67, 95% CI 1.30–2.14, p < 0.0001), obesity (BMI ≥ 28 kg/m^2^) (OR 1.85, 95% CI 1.29–2.66, p = 0.0009) or lower eGFR (< 90 mL/(min·1.73 m^2^)) (OR 1.72, 95% CI 1.33–2.21, p < 0.0001).

**Table 5:** Associations of the TyG quintiles with HTN for different levels of age, BMI, eGFR

Variable	AGE < 55 OR (95% CI) P-value (n = 16,438)	AGE ≥ 55, < 65 OR (95% CI) P-value (n = 17,539)	AGE ≥ 65 OR (95% CI) P-value (n = 9614)	P for interaction	BMI < 18.5 OR (95% CI) P-value (n = 1281)	BMI ≥ 18.5, < 24 OR (95% CI) P-value (n = 19,129)	BMI ≥ 24, < 28 OR (95% CI) P-value (16,758)	BMI ≥ 28 OR (95% CI) P-value (n = 6423)	P for interaction	eGFR < 90 OR (95% CI) P-value (n = 9260)	eGFR ≥ 90 OR (95% CI) P-value (n = 34,331)	P for interaction
N of HTN	4299	7764	6230		259	5902	8137	3995		6018	12,275	
TC, mmol/L				0.4215					0.0339			0.2496
< 1.7	1.0	1.0	1.0		1.0	1.0	1.0	1.0		1.0	1.0	
≥ 1.7, < 2.3	1.23 (1.01, 1.48) 0.0348	1.16 (1.00, 1.34) 0.0470	1.44 (1.18, 1.76) 0.0004		1.05 (0.36, 3.03) 0.9323	1.25 (1.06, 1.48) 0.0081	1.28 (1.11, 1.49) 0.0009	1.19 (0.93, 1.51) 0.1660		1.46 (1.19, 1.79) 0.0003	1.19 (1.06, 1.34) 0.0026	
≥ 2.3	1.13 (0.93, 1.36) 0.2135	1.21 (1.04, 1.40) 0.0111	1.15 (0.93, 1.42) 0.1872		0.10 (0.01, 1.25) 0.0740	0.98 (0.82, 1.17) 0.7960	1.27 (1.10, 1.46) 0.0014	1.36 (1.08, 1.72) 0.0085		1.24 (1.00, 1.54) 0.0477	1.17 (1.04, 1.31) 0.0069	
TC, mmol/L				0.3512					0.5601			0.0882
< 5.2	1.0	1.0	1.0		1.0	1.0	1.0	1.0		1.0	1.0	
≥ 5.2, < 6.2	0.90 (0.77, 1.05) 0.1798	0.98 (0.86, 1.11) 0.7541	0.92 (0.78, 1.09) 0.3530		1.38 (0.69, 2.76) 0.3652	0.91 (0.79, 1.04) 0.1639	0.94 (0.82, 1.07) 0.3449	0.97 (0.78, 1.20) 0.7820		0.88 (0.74, 1.05) 0.1655	0.96 (0.87, 1.06) 0.4072	
≥ 6.2	0.89 (0.72, 1.10) 0.2962	0.85 (0.72, 0.99) 0.0405	1.06 (0.85, 1.33) 0.6183		0.90 (0.36, 2.21) 0.8169	0.83 (0.70, 0.98) 0.0317	0.91 (0.77, 1.08) 0.2750	1.13 (0.85, 1.51) 0.3892		1.08 (0.86, 1.36) 0.5170	0.86 (0.76, 0.98) 0.0197	
HDL-C, mmol/L				0.0368					0.8981			0.1921
≥ 1	1.0	1.0	1.0		1.0	1.0	1.0	1.0		1.0	1.0	
< 1	1.50 (1.23, 1.82) < 0.0001	1.34 (1.14, 1.57) 0.0004	1.03 (0.85, 1.26) 0.7484		1.28 (1.06, 1.54) 0.0091	1.30 (1.14, 1.48) < 0.0001	1.28 (1.10, 1.49) 0.0015	1.25 (0.98, 1.60) 0.0754		1.15 (0.94, 1.41) 0.1785	1.34 (1.19, 1.52) < 0.0001	
LDL-C, mmol/L				0.4126					0.4256			0.1294
< 3.4	1.0	1.0	1.0		1.0	1.0	1.0	1.0		1.0	1.0	
≥ 3.4, < 4.1	1.09 (0.91, 1.30) 0.3674	0.99 (0.87, 1.14) 0.9010	0.93 (0.76, 1.12) 0.4351		1.99 (0.92, 4.30) 0.0798	0.98 (0.85, 1.14) 0.8071	0.96 (0.84, 1.11) 0.6107	1.09 (0.86, 1.39) 0.4817		0.89 (0.73, 1.08) 0.2271	1.04 (0.93, 1.15) 0.5173	
≥ 4.1	0.97 (0.76, 1.23) 0.7791	0.98 (0.82, 1.16) 0.7966	1.20 (0.93, 1.55) 0.1623		1.06 (0.34, 3.26) 0.9206	1.00 (0.83, 1.21) 0.9911	0.96 (0.79, 1.15) 0.6439	1.27 (0.94, 1.73) 0.1246		1.17 (0.90, 1.52) 0.2487	0.98 (0.85, 1.12) 0.7628	
TYG				0.4729					0.1052			0.1328
Q1	1.0	1.0	1.0		1.0	1.0	1.0	1.0		1.0	1.0	
Q2	1.03 (0.83, 1.27) 0.7970	1.04 (0.87, 1.26) 0.6479	1.14 (0.89, 1.46) 0.2946		1.57 (0.79, 3.11) 0.1984	0.97 (0.81, 1.15) 0.6904	1.14 (0.93, 1.40) 0.2199	1.23 (0.84, 1.80) 0.2832		1.13 (0.88, 1.45) 0.3386	1.04 (0.91, 1.20) 0.5523	
Q3	0.98 (0.79, 1.22) 0.8431	1.04 (0.86, 1.25) 0.7139	1.36 (1.07, 1.74) 0.0126		1.03 (0.42, 2.49) 0.9538	1.10 (0.93, 1.31) 0.2629	1.06 (0.87, 1.30) 0.5447	1.42 (0.98, 2.05) 0.0603		1.36 (1.06, 1.74) 0.0146	1.02 (0.89, 1.18) 0.7273	
Q4	1.28 (1.02, 1.60) 0.0308	1.20 (1.00, 1.45) 0.0515	1.67 (1.30, 2.14) < 0.0001		1.68 (0.61, 4.58) 0.3122	1.21 (1.00, 1.45) 0.0496	1.34 (1.10, 1.64) 0.0042	1.85 (1.29, 2.65) 0.0009		1.72 (1.33, 2.21) < 0.0001	1.23 (1.07, 1.42) 0.0037	
Q5	1.18 (0.93, 1.50) 0.1727	1.23 (1.00, 1.50) 0.0448	1.37 (1.05, 1.79) 0.0221		0.70 (0.14, 3.44) 0.6597	0.99 (0.80, 1.22) 0.9168	1.34 (1.09, 1.65) 0.0057	1.85 (1.28, 2.67) 0.0011		1.42 (1.08, 1.87) 0.0115	1.23 (1.06, 1.43) 0.0074	
HbA1c, %				0.5670					0.5270			0.8286
< 6.5	1.0	1.0	1.0		1.0	1.0	1.0	1.0		1.0	1.0	
≥ 6.5	0.83 (0.61, 1.12) 0.2231	1.01 (0.83, 1.24) 0.9083	0.95 (0.75, 1.20) 0.6455		0.51 (0.15, 1.66) 0.2623	0.97 (0.76, 1.22) 0.7763	0.91 (0.74, 1.12) 0.3850	1.11 (0.81, 1.52) 0.5074		1.03 (0.81, 1.29) 0.8299	0.98 (0.84, 1.15) 0.7942	
PBG, mmol/L				0.3262					0.9034			0.3296
< 11.1	1.0	1.0	1.0		1.0	1.0	1.0	1.0		1.0	1.0	
≥ 11.1	0.89 (0.65, 1.21) 0.4452	1.15 (0.93, 1.41) 0.2000	1.15 (0.91, 1.44) 0.2434		0.73 (0.22, 2.47) 0.6141	1.15 (0.91, 1.46) 0.2374	1.10 (0.90, 1.35) 0.3652	1.09 (0.79, 1.50) 0.5916		1.24 (0.99, 1.55) 0.0598	1.08 (0.92, 1.26) 0.3633	

Adjusted for age; center; sex; history of CVDs; history of T2DM; hypoglycemic drugs; SBP; DBP; BMI; ALT; AST; WHR; eGFR; smoking habits; drinking habits

Our study also shows that people with borderline high TG levels (≥ 1.7, < 2.3 mmol/L) were associated with HTN either in all age subgroups, normal and overweight subgroups (BMI ≥ 18.5 kg/m^2^, < 28 kg/m^2^) or both eGFR subgroups. Meanwhile, people with high TG levels (≥ 2.3 mmol/L) were associated with HTN either in the medium age (≥ 55, < 65 years) subgroup, overweight and obesity subgroups (BMI ≥ 28 kg/m^2^) or both eGFR subgroups. People with lower HDL-C levels (< 1 mmol/L) were associated with HTN either in younger and medium age subgroups (< 65 years), normal and overweight subgroups (BMI ≥ 18.5 kg/m^2^, < 28 kg/m^2^) or the higher eGFR (≥ 90 mL/(min·1.73 m^2^)) subgroup. The TC (≥ 6.2 mmol/L) subgroup was associated with HTN only in the medium age subgroup (≥ 55, < 65 years) or with the higher eGFR (≥ 90 mL/(min·1.73 m^2^)) subgroup. A slight association between PBG and HTN was observed only in medium age, overweight and higher eGFR subgroups. However, no apparent association was observed in HbA1c stratified subgroups. The interaction between TG and BMI (p = 0.0339) and between HDL-C and age (p = 0.0368) was significant after adjusting for potential confounders, indicating there would be excess risk due to the additive interaction. When comparing the OR of TyG, lipid and glycemic parameters, the OR of TyG stands out the most, indicating that TyG can be a better discriminator of HTN.

## Discussion

### Main findings

As far as we know, this is the first study to investigate the associations of the TyG index, glycemic, lipid parameters with HTN in a Chinese general population with large sample, multicenter survey. The following are the main findings of this study: (1) The TyG index is significantly associated with HTN and remains significant after LDL-C or HDL-C was well-controlled, and the association of the TyG index with HTN is stronger than lipid or glycemic parameters. (2) HDL-C, TG and PBG are also associated with HTN but are inferior to the TyG index. (3) Further stratification shows that people with a larger BMI (≥ 24 kg/m^2^), older age (≥ 65) and lower eGFR (< 90 mL/(min·1.73 m^2^)) have higher risks of HTN when the TyG index is at a high level (in the fourth and fifth quintiles). Therefore the TyG index is a better discriminator for the risk of HTN compared with lipid and glycemic parameters.

### Glycemic parameters and HTN

It was shown in previous studies that patients with CVDs can benefit from better glycemic control [36, 37]. This research has placed its focus on the average levels and ideal targets of FPG and HbA1c, for the most part. However, this study found only a slight association between HTN and PBG and no apparent association between HTN and HbA1c levels, even in subjects with older age, larger BMI and lower eGFR. Moreover, a slight negative association between HbA1c and HTN was revealed in the male population. There are potential limitations that exist in the assessment of these two glycemic parameters. Although elevated glucose concentration has been treated as a regulable cardiovascular risk factor and a more robust predictor of diabetes than the TyG index [38], FPG only serves as a less effective predictor of cardiovascular outcomes [39]. HbA1c is recommended as the most reliable parameter in the short-term evaluation of glycemic control, but substantial differences have been uncovered between HbA1c and average glycemic level. Especially, similar average glycemic levels could yield considerable discrepancies in HbA1c levels because glucose metabolism and hemoglobin glycation rate might vary corresponding among different individuals [40, 41].

### Lipid parameters and HTN

Dyslipidemia remains as a conventional risk factor for CVD including atherosclerosis, particularly in the general population. Little research has been carried out on the association between lipid parameters and HTN. A 6.4-year follow-up study of 5971 middle-eastern women reported that the predictive value between TG, HDL-C, TG/HDL-C and HTN was most significant among several lipid parameters [42]. Studies on adolescents also reached similar conclusions [43–45]. The results of our study, which show an association between TG, HDL-C and HTN, agree with earlier ones. The sex-specific factors, such as hormone levels, might account for the discrepancies between TG and HTN in different sexes. Reports are limited about the interaction between BMI and TG or HDL-C and age on blood pressure. It is documented that body weight and height decrease and fat mass increase, trunk, and visceral adipose tissue redistribute across higher age [46, 47]. There is a review speculating that increased adipose tissue, which closely relates to TG, contributes to obesity and thus generates an environment in which hypertension can develop [48]. It might account for the interaction between HDL-C and age that low HDL-C is more prevalent in younger and middle age groups; also, longer exposure to environmental and genetic factors might influence the results [49]. Although ACC/AHA and ESC/EAS guidelines have recommended LDL-C to be the most crucial lipid risk factor and therapeutic goal for CVDs [50], LDL-C is not effectively indicative in our study. Moreover, Assmann et al. have proven that the number of clinical events is still alarming regardless of currently desirable LDL-C lowering therapies [51, 52]. In fact, even if LDL-C or HDL-C is well-controlled, a higher TyG index or hypertriglyceridemia is still significantly associated with HTN in our study.

### The mechanisms between the TyG index and HTN

Although the relevant pathophysiological mechanisms responsible for the association between the TyG index and HTN is unclear, several studies suggested the possible mechanism by which IR might affect elevating blood pressure. Theoretically, IR is an essential pathological element involved in metabolic syndrome and a risk factor for elevated blood pressure. Numerous studies have suggested that excess visceral fat represents the cause of metabolic abnormalities leading to increased IR and cardiometabolic risk, including the risk of HTN [53, 54]. Therefore the measurement of IR is valuable in indicating HTN development [53, 54]. In recent years, it has been revealed that the TyG index was closely associated with IR and recommended as a surrogate index of IR [20, 55, 56]. Several studies lend support to the clinical significance of the TyG index for the assessment of atherosclerosis and vascular damage [57, 58]. It has been also reported that the TyG index could be an efficient marker to indicate ischemic stroke, symptomatic CADs and major adverse cardiovascular and cerebral events (MACCEs) [54, 59, 60]. Our findings show that the TyG index, an emerging measurable substitution of IR, is independently associated with HTN after various confounding factors adjustment, which are consistent with previous studies. We have also conducted analysis on other IR markers as shown in Additional file Table S3, demonstrating that the TyG index is a superior discriminator of HTN than other IR markers. IR-compensatory hyperinsulinemia can generate overactivation of the carotid body, bringing on an escalation in sympathetic nervous system activity, further prompting adrenaline and norepinephrine’s secretion, and eventually resulting in cardiac output increases and peripheral vascular resistance [61]. The vascular smooth muscle may be thickened in high concentrated catecholamine, inducing HTN development [62]. Moreover, blood pressure can also be elevated by IR through the activation of the renin-angiotensin-aldosterone system and the increase in endothelin synthesis [63, 64]. Such an increase may contract the blood vessels, decrease the prostacyclin (PGI2) and prostaglandin E2 (PGE2) circulated in vessels which are supposed to be dilated by them [19, 65], and finally proliferate in the vascular smooth muscle, contributing to HTN development.

To be noted, higher eGFR has a close bond to hypertension in the present study. Although the underlying mechanism has not been fully understood, some evidence leads to regard hyperfiltration both a cause and a consequence [66]. Glomerular hyperfiltration can be caused by afferent arteriolar vasodilation, or by efferent arteriolar vasoconstriction owing to activation of the renin-angiotensin-aldosterone system, thus prompting to glomerular hypertension. Thus, early recognition of hyperfiltration will enable clinicians to detect renal diseases and prevent progression of hypertension.

Interestingly, in our study, it is first revealed that the TyG index might be superior to mere glycemic or lipid parameters in associating HTN development. The TyG index is applied in assessing the joint value of TG and FPG because the two parameters are intensively interrelated. Hypertriglyceridemia remains one of the most prevalent abnormalities in T2DM patients, and its association with increased risk of CVDs has been fully demonstrated [67–69]. TG might prompt the formation of atherosclerotic plaque, while glycemic level might be involved in endothelial cells and platelet dysfunction [70–73]. Their values in association with HTN might be better interpreted when they are considered as a whole. Our study shows that the TyG index helps to identify potential risks in individuals who would otherwise be neglected. Clinicians usually put their focus merely on individuals with high FBG or TG. Such conventional clinical practice might possibly miss out on some of the potential risk groups whose FBG and TG are in the normal or borderline ranges.

### Limitations

Our seven-region community-based samples, which representatively demonstrate the distribution of different regions in China, largely and positively influence the research. However, there are still limitations in our study. First, we were not able to directly measure IR in our study population and further compare the surrogate indices with direct markers of IR. Second, because the data of this research was from Chinese elderly individuals, it remains uncertain whether the findings can be applicable to other ethnic groups. Finally, as a feature of the cross-sectional study, only associations, rather than causality, can be established. More prospective studies are needed to identify causal relationship between the TyG index and HTN.

## Conclusion

To conclude, the results of our study reveal a significant association between the TyG index and HTN in Chinese elderly individuals; and it is superior to other lipid profiles and HbA1c and PBG. Therefore we propose that the TyG index could be a more efficient, useful and simple index for the screening and managing of HTN (Additional file 1).

## Supplementary information

**Additional file 1: Table S1.** Association of the TyG index, glycemic, lipid parameters as continuous variables with HTN in total subjects. **Table S2.** Interaction of FBG with TG on hypertension. **Table S3.** Association of TG/HDL and HOMA-IR with HTN in total subjects.

## Data Availability

The datasets used to support this study are not freely available due to participants’ privacy protection.

## Abbreviations

ALT: Alanine transferase
AST: Aspartate transferase
BMI: Body mass index
CHD: Coronary heart disease
CVD: Cardiovascular disease
CI: Confidence interval
DBP: Diastolic blood pressure
eGFR: Estimated glomerular filtration rate
FBG: Fasting plasma glucose
GGT: Gamma-glutamyl transferase
HbA1c: Glycosylated hemoglobin
HDL-C: High-density lipoprotein cholesterol
HR: Heart rate
HTN: Hypertension
IR: Insulin resistance
LDL-C: Low-density lipoprotein cholesterol
MACCEs: Major adverse cardiovascular and cerebral events
OGTT: Glucose tolerance test
OR: Odds ratio
PBG: 2 h post-load blood glucose
PGE2: Prostaglandin E2
PGI2: Prostacyclin
SBP: Systolic blood pressure
TC: High cholesterol
TG: Triglyceride
TyG: Triglyceride-glucose
T2DM: Type 2 diabetes
WHR: Waist circumference/hip circumference
