# Supplementary material for: A high triglyceride glucose index is more closely associated with hypertension than lipid or glycemic parameters in elderly individuals: a cross-sectional survey from the Reaction Study
Source: Cardiovasc Diabetol. 2020 Jul 14;19:112. doi: 10.1186/s12933-020-01077-6 (PMC7362407; doi:10.1186/s12933-020-01077-6)
Supplement: Supplementary file 1 — Additional file 1: Table S1. Association of the TyG index, glycemic, lipid parameters as continuous variables with HTN in total subjects. Table S2. Interaction of FBG with TG on hypertension. Table S3. Association of TG/HDL and HOMA-IR with HTN in total subjects. [file 12933_2020_1077_MOESM1_ESM.docx]

**Table S1. Association of the TyG index, glycemic, lipid parameters as continuous variables with HTN in total subjects**

| **_Variable_** | **_Non-adjusted_**  **_OR (95% CI) P-value_** | **_Adjust I_**  **_OR (95% CI) P-value_** | **_Adjust II_**  **_OR (95% CI) P-value_** |
| --- | --- | --- | --- |
| **_TG, mmol/L_** | _1.91 (1.84, 1.98) <0.0001_ | _1.85 (1.78, 1.92) <0.0001_ | _1.18 (__1.10, 1.27) <0.0001_ |
| **_TC, mmol/L_** | _1.96 (1.81, 2.12) <0.0001_ | _1.78 (1.64, 1.93) <0.0001_ | _0.88 (0.74, 1.04) 0.1319_ |
| **_HDL-C, mmol/L_** | _0.62 (0.57, 0.66) <0.0001_ | _0.66 (0.61, 0.71) <0.0001_ | _0.68 (0.58, 0.79) <0.0001_ |
| **_LDL-C, mmol/L_** | _1.54 (1.45, 1.63) <0.0001_ | _1.40 (1.32, 1.49) <0.0001_ | _0.89 (0.79, 1.00) 0.0553_ |
| **_TyG_** | _2.53 (2.42, 2.64) <0.0001_ | _2.29 (2.19, 2.40) <0.0001_ | _1.18 (__1.08, 1.28)_ _<0.0001_ |
| **_HbA1c, %_** | _8.47 (7.34, 9.77) <0.0001_ | _4.03 (3.48, 4.66) <0.0001_ | _0.81 (0.56, 1.16) 0.2521_ |
| **_PBG, mmol/L_** | _3.14 (2.98, 3.30) <0.0001_ | _2.37 (2.25, 2.50) <0.0001_ | _1.35 (1.19, 1.54) <0.0001_ |

_Model 0: Adjusted for no confounding factors;_

_Model 1: Adjusted for age and gender;_

_Model 2: age; center; sex; history of CVDs; history of T2DM; hypoglycemic drugs; SBP; DBP; BMI; ALT; AST; WHR; eGFR; smoking habits, drinking habits._

**Table S2. Interaction of FBG with TG on hypertension.**

| _Variable_ | _Non-adjusted_  _OR (95% CI) P-value_ | _Adjust I_  _OR (95% CI) P-value_ | _Adjust II_  _OR (95% CI) P-value_ |
| --- | --- | --- | --- |
| _TG, mmol/L_ |  |  |  |
| _<1.7_ | _1.26 (1.24, 1.28) <0.0001_ | _0.94 (0.90, 0.98) 0.0026_ | _0.95 (0.91, 1.00) 0.0303_ |
| _≥1.7, <2.3_ | _1.17 (1.14, 1.20) <0.0001_ | _0.92 (0.87, 0.98) 0.0042_ | _0.89 (0.83, 0.95) 0.0009_ |
| _≥2.3_ | _1.08 (1.06, 1.11) <0.0001_ | _0.94 (0.90, 0.99) 0.0112_ | _0.94 (0.89, 1.00) 0.0465_ |
| _P for interaction_ | _<0.0001(<0.0001 #)_ | _0.8070(0.8635 #)_ | _0.2634(0.7482 #)_ |

_Model 0: Adjusted for no confounding factors;_

_Model 1: Adjusted for_ _age; center; sex; history of CVDs; history of T2DM; hypoglycemic drugs; SBP; DBP; BMI; ALT; AST; WHR; eGFR; smoking habits, drinking habits._

_Model 2: age; center; sex; history of CVDs; history of T2DM; hypoglycemic drugs; SBP; DBP; BMI; ALT; AST; WHR; eGFR; smoking habits, drinking habits and the interaction terms for following variables: history of T2DM; hypoglycemic drugs._

_# interaction trend test (treat TG as continuous)_

**Table S3. Association of TG/HDL and HOMA-IR with HTN in total subjects**

| **_Variable_** | **_Non-adjusted_**  **_OR (95% CI) P-value_** | **_Adjust I_**  **_OR (95% CI) P-value_** | **_Adjust II_**  **_OR (95% CI) P-value_** |
| --- | --- | --- | --- |
| **_TG/HDL-c_** | _1.67 (1.62, 1.72) <0.0001_ | _1.53 (1.48, 1.58) <0.0001_ | _1.18 (1.11, 1.26) <0.0001_ |
| **_HOMA-IR_** | _2.40 (2.32, 2.49) <0.0001_ | _2.38 (2.28, 2.48) <0.0001_ | _1.37 (1.27, 1.48) <0.0001_ |

_Model 0: Adjusted for no confounding factors;_

_Model 1: Adjusted for age and gender;_

_Model 2: age; center; sex; history of CVDs; history of T2DM; hypoglycemic drugs; SBP; DBP; BMI; ALT; AST; WHR; eGFR; smoking habits, drinking habits._
